# Supplementary material for: Towards novel herbicide modes of action by inhibiting lysine biosynthesis in plants
Source: eLife. 2021 Jul 27;10:e69444. doi: 10.7554/eLife.69444 (PMC8341977; doi:10.7554/eLife.69444)
Supplement: Supplementary file 1. [file elife-69444-supp1.docx]

|  | MBDTA-1 | MBDTA-2 |
| --- | --- | --- |
| Molecular mass (Da) | 293.3 | 293.3 |
| clog*P* | 1.96 | 1.96 |
| clog*S* | -2.375 | -2.379 |
| pKa | 3.115 | 3.143 |
| tPSA | 83.91 | 83.91 |
| #H-bond donors | 1 | 1 |
| #H-bond acceptors | 6 | 6 |
